# Hydrate of Chloral Poisoning

**Published:** 1876-11

**Authors:** J. H. W. Meyer

**Affiliations:** Cook County Hospital


### HYDRATE OF CHLORAL POISONING.

By J. H. W. MEYER, M.D., Cook County Hospital.

On the 7th of August, 1876, Miss Lizzie King, patient in Cook County Hospital, appropriated the ward bottle containing, in an eight ounce solution, one ounce of hydrate of chloral and two ounces of bromide of potassium. This she took with suicidal intent, leaving only a drachm or two of the solution in the bottle. Dreading the result she tried to keep awake. When she took the medicine she was in the bath room, where, some ten minutes after, a patient noticed her drowsiness. In about five minutes more another patient found her fast asleep. On lifting her up, the empty ward bottle was found about her person, and the case reported at the office.

I found her in a comatose condition, breath smelling strongly of chloral, pupils contracted, extremities cold, pulse less at the wrist, heart's action very feeble, reflex irritability almost lost, and general sensation diminished. All ordinary efforts failed to awaken her.

I administered thirty grains of sulphate of zinc followed by draughts of warm water containing mustard. As no efforts at vomiting were noticeable, I irritated the palate by the introduction of the finger, reaching down as far as possible; still no result. The stomach tube was now introduced, and, connecting it through rubber tubing with a bag full of warm water (the bag being a part of Dr. Potter's universal syringe), the stomach was filled with the water by holding the bag a foot or more above the head, and again emptied by lowering it. As the bag was emptied the water smelled of chloral; therefore this process was repeated about four times, till the water on its return had no smell of chloral. She then made an effort to vomit. Before the tube was removed some whisky was introduced through it into the stomach. During this time her condition had somewhat improved. Pulse could be felt at the wrist, and the pupils began to dilate. As she was still unconscious, recourse was had to the cold douche, the water being poured from a height of several feet upon the head and abdomen. Soon she tried to avoid the water, but still slept on. After this treatment had been continued about ten minutes she so far aroused as to beg to be let alone, but still slept. After this she was rubbed vigorously with coarse towels till the skin became warm, when she was put to bed, a drachm of the aromatic spirits of ammonia administered and efforts continued to awaken her. After this she opened her eyes, but soon relapsed into her former condition. I then applied the battery, passing strong currents through the extremities. This so far aroused her, that she attempted to answer questions, in a very incoherent manner, however. After about three hours from the time she was found asleep, I considered her out of danger; but as she would still fall asleep when undisturbed, I repeated the dose of spirits of ammonia, and charged the nurse to watch her closely and to arouse her at short intervals by rubbing and shaking. On the morning of the 8th she felt about as well as she did before taking the medicine, but remembers nothing of the treatment.

Case II. Mr. S. was found in his bed on the morning of the 8th of August, 1876, in an insensible condition. An empty bottle, labeled hydrate of chloral, being found near, he was sent to Cook County Hospital as a case of chloral poisoning. On his arrival he was found comatose, but no other alarming symptoms were present. No efforts were made to empty the stomach, the chloral having undoubtedly been taken several hours previously and, being rapidly absorbed, we did not suppose any present in the stomach. During the day stimulants were administered, such as whisky and aromatic spirits of ammonia, under belief that the effects of the chloral would gradually pass away, as it was eliminated by the lungs and other emunctories. It was impossible to get any history from him during the day. Being still in nearly the same condition in the evening recourse was had to energetic treatment, beginning with flagellations. This aroused him so that he answered several questions, stating that he had taken half an ounce of hydrate of chloral with intent to kill. Could not then remember how long since he had taken it. In answering questions he seemed to forget the answer, but on urging, after several trials, would finish a sentence, falling asleep after uttering a few words. To avoid injuring the skin the cold shower bath was substituted for the flagellation, which decidedly improved his condition, so that with assistance he walked from the bath room to his bed. As he was inclined to relapse and to secure the advantage gained, recourse was had to the battery, which stimulated him very materially. All day it was impossible to get him to swallow any nourishment, but now he took a cup full of milk punch. Being a great deal better he was left to rest for the night.

On the morning of the 9th he was still under the influence of the chloral, and answering questions slowly. From this time on his recovery was satisfactory. On the 10th he stated that he had taken the chloral on the evening of the 7th, at eight o'clock, and that he did not remember anything from the time he went to sleep on the 7th till he was aroused the evening of the 8th by the flagellations, bath and battery.

